# Associated health and social determinants of mobile populations across HIV epidemic gradients in Southern Africa

**DOI:** 10.1016/j.jmh.2021.100038

**Published:** 2021-03-26

**Authors:** Esteban Correa-Agudelo, Hae-Young Kim, Godfrey N. Musuka, Zindoga Mukandavire, Adam Akullian, Diego F. Cuadros

**Affiliations:** a Department of Geography and Geographic Information Science, University of Cincinnati, Cincinnati, OH, 45221, USA; b Health Geography and Disease Modeling Laboratory, University of Cincinnati, Cincinnati, USA; c Africa Health Research Institute, KwaZulu-Natal, South Africa; d KwaZulu-Natal Research Innovation and Sequencing Platform (KRISP) KwaZulu-Natal, South Africa; e Department of Population Health, New York University Grossman School of Medicine, USA; f ICAP at Columbia University, Harare, Zimbabwe; g Centre for Data Science, Coventry University, UK; h School of Computing, Electronics and Mathematics, Coventry University, UK; i Institute for Disease Modeling, Global Good Fund, Bellevue, Washington, USA; j Department of Global Health, University of Washington, Seattle, Washington, USA

**Keywords:** Disease mapping, Health determinants, Mobile population, Southern Africa, Spatial epidemiology

## Abstract

**Background:**

Growing travel connectivity and economic development have dramatically increased the magnitude of human mobility in Africa. In public health, vulnerable population groups such as mobile individuals are at an elevated risk of sexually transmitted diseases, including HIV.

**Methods:**

The population-based Demographic Health Survey data of five Southern African countries with different HIV epidemic intensities (Angola, Malawi, South Africa, Zambia, and Zimbabwe) were used to investigate the association between HIV serostatus and population mobility adjusting for socio-demographic, sexual behavior and spatial covariates.

**Results:**

Mobility was associated with HIV seropositive status only in Zimbabwe (adjusted odds ratio [AOR] = 1.37 [95% confidence interval [CI]: 1.01–1.67]). These associations were not significant in Angola, Malawi, South Africa, and Zambia. Females had higher odds of mobility than males in Zimbabwe (AOR = 1.37, CI: 1.10–1.69). The odds of mobility decreased with age in all five countries.

**Conclusions:**

Our findings highlight the heterogeneity of the social and health determinants of mobile populations in several countries with different HIV epidemic intensities. Effective interventions using precise geographic focus combined with detailed attribute characterization of mobile populations can enhance their impact especially in areas with high density of mobile individuals and high HIV prevalence.

## 1 Introduction

Growing travel connectivity and economic development have dramatically increased the magnitude of population mobility in Africa (Zlotnik, 2006). In public health, vulnerable population groups such as mobile individuals are at an elevated risk of sexually transmitted diseases, including HIV. Several studies have shown that mobile population may contribute to a higher risk of both HIV acquisition and transmission. For example, in Malawi, individuals living with HIV migrate at higher rates than those not living with HIV (Anglewicz and Anglewicz, 2012). In South Africa, migrant populations from a rural community experienced an increased risk of HIV acquisition (Lurie et al., 2003). Labor related mobility to agricultural estates and mines among men had been identified as a key driver of the HIV epidemic in Zambia (International Organization for Migration, 2006). In Zimbabwe, a country with one of the worst HIV epidemics, travel and international trade have been associated with high HIV prevalence at the national borders (Wilson, 2000). Similarly, mobile women residing along Kenya's lakeshore fishing communities who participate in commercial sex experienced an increased risk of HIV acquisition (Camlin et al., 2014).

Although several studies have shown the evidence of link between population mobility and HIV risk using cohort data at the community level, limited studies have examined how demographic and spatial factors are associated with mobile populations in different HIV epidemic settings using population-based national survey data. Therefore, the current research points to the necessity to i) assess the socioeconomic and health determinants associated with mobile populations in heterogeneous HIV epidemics at national level, and ii) to identify the spatial structure of mobile population in relation to the HIV prevalence in each country. In this study, we examined the association between population mobility and HIV serostatus using the population-based Demographic Health Survey (DHS) of five Southern African countries with different HIV epidemic intensities (Angola, Malawi, South Africa, Zambia, and Zimbabwe) and examined the association between population mobility and HIV serostatus.

## 2 Materials and methods

### 2.1 Study area and data sources

The study area focuses on five geographically contiguous countries with differential national HIV prevalence in Southern Africa. In 2019, the prevalence of HIV was 2.0% in Angola, 9.2% in Malawi, 20.0% in South Africa, 11.3% in Zambia, and 12.7% in Zimbabwe (UNAIDS). Data were derived from the most recent DHS that included HIV biomarkers and georeferenced data as following: Angola 2015–2016 (Instituto Nacional de Estatística - INE/Angola 2017), Malawi 2015–2016 (National Statistical Office/Malawi 2017), South Africa 2016 (National Department of Health 2019), Zambia 2018 (Zambia Statistics Agency ZSA 2020) and Zimbabwe 2015 (Zimbabwe National Statistics Agency 2016). DHS surveys encompass individuals and household level evaluation indicators in health, nutrition, socioeconomic, and HIV serostatus over time. The DHS survey design is a two-stage sampling procedure through a set of defined locations (primary sample units or PSU) statistically weighted to control for sample biases (ICF International 2012). A total of 627 PSUs were selected for Angola, 850 PSUs for Malawi, 750 for South Africa, 545 PSUs for Zambia, and 400 PSUs for Zimbabwe. All surveys covered 108,401 adults in reproductive age (15–59 years). 20,063 individuals in Angola, 32,040 individuals in Malawi, 12,132 individuals in South Africa, 25,815 individuals in Zambia, and 18,351 individuals in Zimbabwe. This study was limited to a sample of adults who had valid mobility status and HIV testing data and complete data for all covariates (See Figure A.2), resulting in a total of 8536 individuals (4961 women and 3575 men) for Angola, 10,827 individuals (5769 women and 5058 men) in Malawi, 3327 individuals (1389 women and 1938 men) in South Africa, 17,966 individuals (9560 women and 8406 men) in Zambia, and 10,898 individuals (6084 women and 4814 men) in Zimbabwe. See Appendix A for further details related to the inclusion criteria.

### 2.2 Study variables

The primary outcome of interest was mobility status derived from the time of residence in a place. We used the DHS variable *“Years lived in the place of residence”* as the measure of mobility, which was grouped into two categories: *Mobile* category included people who recently moved and had lived in the place of current residence for less than a year at the time of DHS survey. *Non-mobile* category included the residents who had resided for one or more than a year in the place of current residence. HIV serostatus estimated from the blood test result was considered as an independent covariate. HIV testing was performed using dried blood spots samples among women and men after written informed consent was obtained. HIV serostatus was determined by testing with the enzyme-linked immunosorbent assay (ELISA) Vironostika Uniform 2 Ag/AB manufacturer. Further details related to the DHS methodology, study design, and data can be found elsewhere (ICF International, 2012).

Socioeconomic, demographic, sexual behavior, and spatial covariates of the mobile population were characterized in each country. Sociodemographic covariates included: age, education, wealth index, health insurance status, marital status, and type of residence. For sexual behavior, we included: condom used last time of sexual intercourse, the history of having any sexually transmitted infections (STI) in the last 12 months, HIV testing history, number of partners in the last 12 months, and total lifetime number of sexual partners. Additionally, we obtained DHS geospatial covariates for each PSU to assess proximity to national borders using the geodesic distance to the nearest international borders, and travel time in minutes to nearest high-density urban centers. Covariates were classified using appropriate levels: age groups (15–24, 25–34, 35–44, 45+), education (no education/primary, secondary or higher), wealth index combined (poorer/poorest or middle/richer/richest), health insured (yes or no), previous STI (yes or no), marital status (never in union, married/living with a partner, or divorced/separated/widowed), type of residence (urban or rural), having any STI in last 12 months (yes or no), prior HIV testing (yes or no), number of partners in last 12 months (0–1 sex partner or 2+ partners), total lifetime sex partners (0–1 sex partner or 2+ partners), proximity to national borders (<50 km, 50 km - 100 km, 100km+), and travel time (< 1hr, 1hr – 2 hrs, 2hrs+).

### 2.3 Statistical analysis

All covariates were selected according to an evidence synthesis process of relevant references. Then, an implied graph (IG) was built to infer causal effects to the observational data. Next, backdoor criterion was performed to remove open paths, check for colliders and overcontrol in the implied graph (See Appendix A). Complementary to the evidence synthesis and DAG analysis, we performed a Variance Inflated Factor (VIF) to account for multicollinearity. All variables less than five (VIF<5.0) were included in the final adjusted model (See Appendix B). Since DHS surveys are collected in different years, each country was analyzed individually. For interpretation, we have assumed no temporal trend between all five datasets.

Logistic regression models were fitted to assess the association between mobility and the selected covariates. DHS two-stage cluster sampling procedure was considered to correctly estimate sampling errors through all fitted models. Since there is an expectation that the two variables might be related in space (percentage of mobile individuals and HIV prevalence), continuous surface maps of mobile population and HIV prevalence were generated. Each map was normalized and classified with an equal interval scheme. Finally, a bivariate palette was designed to visually depict all possible combinations of the proportion of mobile individuals and HIV prevalence in all countries. R programming environment (R Core Team 2018) was used to generate maps and models (See Appendix B and C in Supplementary Materials).

## 3 Results

### 3.1 Population profile

Baseline characteristics differentiated by mobility status for each country are included in Table 1. The overall proportion of the population classified as mobile individuals was 6% across the five countries. Women migrated more than men in Malawi (60% vs 40%; *p* = 0.014), Zambia (61% vs 39%; *p* = 0.004), and Zimbabwe (61% vs 39%; *p* = 0.003). On average, mobile individuals were younger compared to non-mobile individuals in all countries: Angola (22.4 years vs 29.2 years; *p*<0.001), Malawi (26.1 years vs 30.5 years; *p*<0.001), South Africa (27.8 years vs 31.8 years; *p*<0.001), Zambia (27.7 years vs 31.7 years; *p*<0.001), and Zimbabwe (26.1 years vs 31.7 years; *p*<0.001). Mobile individuals with middle and high wealth index were more common in Malawi (70% vs 61%; *p* = 0.006), Zambia (77% vs 60%; *p*<0.001) and Zimbabwe (72% vs 64; *p* = 0.002), compared to South Africa (44% vs 56%; *p* = 0.017). The average proportion of insured mobile individuals was 5.4% in all countries, ranging from 3% in Zambia to 7% in Angola. The proportion of never being in union was higher among mobile compared to non-mobile population in Angola, Zambia and Zimbabwe, with the lowest difference in Zambia (27% vs 22%; *p*<0.001), and the largest in Angola (46% vs 31%; *p*<0.025), respectively. Mobile individuals were more likely living in urban residences in Malawi (40% vs 16%; *p*<0.001), and Zambia (59% vs 39%; *p*<0.001).

**Table 1:** Population characteristics by mobility status.

		Angola					Malawi					South Africa				
		Mobile		Permanent resident		*p-value*	Mobile		Permanent resident		*p-value*	Mobile		Permanent resident		*p-value*
N/weighted	No. (%)	109	(1)	8427	(99)		657	(6)	10,170	(94)		211	(6)	3115	(94)	
HIV sero-status	No. (%)					0.833					0.368					0.162
HIV-		107	(98)	8260	(98)		583	(89)	9168	(90)		150	(71)	2399	(77)	
HIV+		2	(2)	167	(2)		74	(11)	1002	(10)		61	(29)	716	(23)	
Gender						0.134					0.014					0.229
Male		60	(55)	3515	(42)		261	(40)	4797	(47)		78	(37)	1311	(42)	
Female		49	(45)	4912	(58)		396	(60)	5373	(53)		133	(63)	1805	(58)	
Age (years)	mean (se)	22.4	(0.66)	29.2	(0.17)	<0.001	26.1	(0.40)	30.5	(0.13)	<0.001	27.8	(0.71)	31.8	(0.25)	<0.001
Age groups						<0.001					<0.001					<0.001
15–24		79	(73)	3285	(39)		338	(51)	3306	(33)		82	(39)	920	(30)	
25–34		26	(24)	2653	(31)		224	(34)	3395	(33)		88	(42)	1024	(33)	
35–44		3	(3)	1704	(20)		76	(11)	2460	(24)		35	(17)	738	(24)	
45+		0	(0)	785	(9)		19	(3)	1009	(10)		6	(3)	434	(14)	
Education	No. (%)					0.460					<0.001					0.446
Higher		3	(2)	482	(6)		49	(7)	366	(4)		16	(7)	324	(10)	
No education /Primary		62	(57)	4244	(50)		343	(52)	7348	(72)		37	(17)	462	(15)	
Secondary		44	(41)	3701	(44)		265	(40)	2456	(24)		159	(75)	2329	(75)	
Wealth index combined	No. (%)					0.774					0.006					0.017
Middle/Richer/ Richest		73	(68)	5367	(64)		460	(70)	6233	(61)		93	(44)	1759	(56)	
Poorest/Poorer		35	(32)	3060	(36)		197	(30)	3936	(39)		118	(56)	1356	(44)	
Health insured	No. (%)					0.970					<0.001					<0.001
No		101	(93)	7857	(93)		627	(95)	9986	(98)		201	(95)	2726	(87)	
Yes		8	(7)	570	(7)		30	(5)	184	2		10	(5)	389	(13)	
Marital status	No. (%)					0.025					0.172					0.158
Never in union		50	(46)	2573	(31)		113	(17)	1531	(15)		100	(47)	1700	(55)	
Married/Living with a partner		51	(46)	5406	(64)		492	(75)	8029	(79)		93	(44)	1259	(40)	
Divorced/Separated/ Widowed		9	(8)	449	(5)		52	(8)	609	(6)		18	(9)	156	(5)	
Type of residence	No. (%)					0.578					<0.001					0.357
Urban		83	(76)	5698	(68)		262	(40)	1582	(16)		140	(66)	1937	(62)	
Rural		26	(24)	2729	(32)		396	(60)	8588	(84)		71	(34)	1178	(38)	
Condom used last time	No. (%)					0.156					0.177					0.99
No		79	(72)	6918	(82)		516	(78)	8284	(81)		110	(52)	1627	(52)	
Yes		30	(28)	1510	(18)		141	(22)	1886	(19)		101	(48)	1488	(48)	
Previous STI	No. (%)					0.062					0.477					0.498
No		107	(98)	7943	(94)		636	(97)	9906	(97)		203	(96)	2966	(95)	
Yes		2	(2)	485	(6)		21	(3)	264	(3)		8	(4)	149	(5)	
Prior HIV testing	No. (%)					0.027					0.690					0.099
No		80	(73)	4550	(54)		99	(15)	1453	(14)		21	(10)	451	(14)	
Yes		29	(27)	3877	(46)		558	(85)	8716	(86)		190	(90)	2664	(86)	
Sex partners in last 12 months	No. (%)					0.584					0.618					0.244
0–1		101	(93)	7574	(90)		604	(92)	9274	(91)		178	(84)	2722	(87)	
2+		8	(7)	853	(10)		53	(8)	896	(9)		33	(16)	393	(13)	
Total lifetime sex partners	No. (%)					0.842					0.579					0.324
0–1		30	(27)	2413	(29)		204	(31)	3274	(32)		31	(15)	572	(18)	
2+		79	(73)	6014	(71)		454	(69)	6896	(68)		180	(85)	2543	(82)	
Proximity to National Borders (Km)	No. (%)					0.112					<0.001					0.654
<50 Kms		60	(55)	4560	(54)		388	(59)	7282	(72)		57	(27)	960	(31)	
50 Kms - 100 Kms		24	(22)	717	(9)		269	(41)	2887	(28)		37	(17)	534	(17)	
100 Kms +		24	(22)	3150	(37)							118	(56)	1621	(52)	
Travel time to nearest city (Hours)	No. (%)					0.203					<0.001					0.075
2 hrs +		7	(6)	1703	(20)		32	(5)	876	(9)		4	(2)	118	(4)	
1 hr - 2 hrs		23	(21)	869	(10)		197	(30)	3987	(39)		30	(14)	567	(18)	
< 1 hr		79	(73)	5855	(69)		428	(65)	5307	(52)		177	(84)	2430	(78)	
< 1 hr														*(Continued on next page)*

**Table 1:** (*Continued*)

		Zambia					Zimbabwe									
		Migrant		Non-Migrant		*P*	Migrant		Non-Migrant		*P*					
N/weighted	No. (%)	1682	(9)	16,284	(91)		778	(7)	10,122	(93)						
HIV sero-status	No. (%)					0.153					0.666					
HIV-		1445	(86)	14,249	(87)		651	(84)	8538	(84)						
HIV+		237	(14)	2036	(13)		127	(16)	1583	(16)						
Gender						0.004					0.003					
Male		656	(39)	7750	(48)		300	(39)	4514	(45)						
Female		1026	(61)	8534	(52)		477	(61)	5607	(55)						
Age	mean (se)	27.7	(0.27)	31.7	(0.11)	<0.001	26.1	(0.32)	31.7	(0.13)	<0.001					
Age groups						<0.001					<0.001					
15–24		732	(44)	4744	(29)		394	(51)	2418	(24)						
25–34		616	(37)	5353	(33)		266	(34)	3996	(39)						
35–44		260	(15)	4098	(25)		91	(12)	2749	(27)						
45+		74	(4)	2089	(13)		27	(3)	958	(9)						
Education	No. (%)					<0.001					<0.001					
Higher		162	(10)	1108	(7)		48	(6)	993	(10)						
No education /Primary		692	(41)	8326	(51)		185	(24)	2791	(28)						
Secondary		827	(49)	6850	(42)		545	(70)	6337	(63)						
Wealth index combined	No. (%)					<0.001					0.002					
Middle/Richer/ Richest		1300	(77)	9841	(60)		557	(72)	6455	(64)						
Poorest/Poorer		382	(23)	6443	(40)		220	(28)	3666	(36)						
Health insured	No. (%)					0.502					<0.001					
No		1633	(97)	15,870	(97)		723	(93)	8848	(87)						
Yes		49	(3)	415	(3)		55	(7)	1273	(13)						
Marital status	No. (%)					<0.001					<0.001					
Never in union		449	(27)	3537	(22)		178	(23)	1316	(13)						
Married/Living with a partner		1086	(65)	11,644	(72)		478	(61)	8111	(80)						
Divorced/Separated/ Widowed		147	(9)	1104	(7)		122	(16)	695	(7)						
Type of place	No. (%)					<0.001					0.677					
Urban		992	(59)	6397	(39)		272	(35)	3664	(36)						
Rural		690	(41)	9887	(61)		505	(65)	6458	(64)						
Condom used last time	No. (%)					0.018					<0.001					
No		1301	(77)	13,307	(82)		543	(70)	7786	(77)						
Yes		381	(23)	2977	(18)		235	(30)	2336	(23)						
Previous STI	No. (%)					0.115					0.448					
No		1614	(96)	15,767	(97)		755	(97)	9880	(98)						
Yes		68	(4)	518	(3)		22	(3)	242	(2)						
Prior HIV testing	No. (%)					0.192					0.088					
No		153	(9)	1682	(10)		136	(18)	1460	(14)						
Yes		1529	(91)	14,603	(90)		641	(82)	8662	(86)						
Sex partners in last 12 months	No. (%)					0.674					0.017					
0–1		1515	(90)	14,600	(90)		684	(88)	9175	(91)						
2+		167	(10)	1684	(10)		94	(12)	946	(9)						
Total lifetime sex partners	No. (%)					0.954					0.384					
0–1		401	(24)	3863	(24)		311	(40)	4240	(42)						
2+		1281	(76)	12,422	(76)		466	(60)	5881	(58)						
Proximity to National Borders (Km)	No. (%)					<0.001					0.004					
<50 Kms		667	(41)	7341	(46)		149	(19)	1969	(19)						
50 Kms - 100 Kms		671	(41)	5129	(32)		121	(16)	2241	(22)						
100 Kms +		303	(18)	3509	(22)		507	(65)	5912	(58)						
Travel time to nearest city (Hours)	No. (%)					<0.001					0.02					
2 hrs +		192	(12)	3482	(22)		136	(17)	2245	(22)						
1 hr - 2 hrs		248	(15)	3693	(23)		180	(23)	2564	(25)						
< 1 hr		1201	(73)	8814	(55)		462	(59)	5313	(52)						

Weighted frequencies and column percentages shown.

Ever being tested for HIV among mobile individuals was 85% in Malawi, 90% in South Africa, 91% in Zambia, and 82% in Zimbabwe, whereas only 27% of the mobile population in Angola reported being tested for HIV. Most mobile populations reported no previous STI (≥96%) and less than two sexual partners (84%) in the last 12 months. More than 60% of the mobile individuals reported having two or more lifetime sexual partners in all countries (73% in Angola, 69% in Malawi, 85% in South Africa, 76% in Zambia, and 60% in Zimbabwe). Mobile individuals moved closer than 50 km to the national borders of Angola (55%) and Malawi (59%). The proportion of mobile individuals with less than one hour of travel time to the nearest city was ≥59% in all countries.

### 3.2 Health determinants in mobile individuals

Table 2 summarizes the results from the adjusted model for the association between mobility status and the covariates in all countries. Mobility was associated with HIV seropositive status only in Zimbabwe (adjusted odds ratio [AOR] = 1.37 [95% confidence interval [CI]: 1.01–1.67]). These associations were not significant in Angola (AOR = 1.45, CI: 0.32–6.58) Malawi (AOR = 1.16, CI: 0.80–1.68), South Africa (AOR = 1.28, CI: 0.79–2.06), and Zambia (AOR = 1.04, CI: 0.85–1.28). Females had higher odds of mobility than males in Zimbabwe (AOR = 1.37, CI: 1.10–1.69). Similarly, odds of movement between places decreased with age in all countries (Table 2). Education was not associated with being mobile in all countries. Low wealth index combined was a protective factor for mobility in Zambia (AOR = 0.67, CI: 0.54–0.83), and Zimbabwe (AOR = 0.65, CI: 0.50–0.85). Mobile individuals had higher odds of being divorced, separated, or widowed in all countries. Mobile individuals had lower odds of moving to rural places in Malawi (AOR = 0.36, CI: 0.26–0.49), and Zambia (AOR = 0.70, CI: 0.55–0.90), but higher odds in Zimbabwe (AOR = 1.56, CI: 1.16–2.10). Mobile individuals had higher odds of having two or more total lifetime sexual partners in Zambia (AOR = 1.24, CI: 1.05–1.46), and Zimbabwe (AOR = 1.27, CI: 1.04–1.57). Condom usage, HIV testing history, previous STI, or the number of sex partners in last 12 months were not associated with being mobile. Conversely, mobile individuals had higher odds of establishing in areas close to national borders between 50 km to 100 km in Angola (AOR = 3.08, CI: 1.13–8.40) and Zambia (AOR = 1.30, CI: 1.05–1.62), but not in Zimbabwe (AOR = 0.68, CI: 0.50–0.92). Lastly, mobile individuals had higher odds of establishing near to main cities in Zambia (AOR = 1.64, CI: 1.29–2.09) and Zimbabwe (AOR = 1.48, CI: 1.08–2.04).

**Table 2:** Adjusted odds ratios showing associations between mobility status and control variables in Angola, Malawi, South Africa, Zambia, and Zimbabwe.

	Angola		Malawi		South Africa	
	AOR (95% CI)		AOR (95% CI)		AOR (95% CI)	
HIV sero-status						
HIV-	Ref		Ref		Ref	
HIV+	1.45	(0.32, 6.58)	1.16	(0.80, 1.68)	1.28	(0.79, 2.06)
Gender						
Male	Ref	Ref	Ref			
Female	0.50	(0.24, 1.05)	1.24	(0.95, 1.63)	1.21	(0.79, 1.84)
Age groups						
15–24	Ref	Ref	Ref			
25–34	**0.38**	**(0.18, 0.80)**	**0.45**	**(0.35, 0.59)**	**0.65**	**(0.41, 1.02)**
35–44	**0.06**	**(0.02, 0.19)**	**0.22**	**(0.15, 0.32)**	**0.31**	**(0.17, 0.56)**
45+	**0.01**	**(0.00, 0.06)**	**0.15**	**(0.08, 0.29)**	**0.08**	**(0.03, 0.19)**
Education						
Higher	Ref	Ref	Ref			
No education /Primary	2.43	(0.59, 10.03)	0.58	(0.32, 1.06)	1.50	(0.66, 3.41)
Secondary	1.24	(0.29, 5.33)	0.99	(0.55, 1.81)	1.04	(0.52, 2.09)
Wealth index combined						
Middle/Richer /Richest	Ref	Ref	Ref			
Poorest/Poorer	0.82	(0.37, 1.78)	0.99	(0.73, 1.33)	1.67	0.99, 2.82)
Health insured						
No	Ref	Ref	Ref			
Yes	1.24	(0.28, 5.46)	1.47	(0.84, 2.58)	**0.51**	**(0.26, 0.97)**
Marital status						
Never in union	Ref	Ref	Ref			
Married/Living with a partner	1.52	(0.70, 3.32)	**2.12**	**(1.40, 3.23)**	**1.93**	**(1.22, 3.05)**
Divorced/Separated /Widowed	**2.83**	**(1.10, 7.28)**	**2.53**	**(1.51, 4.24)**	**3.82**	**(1.69, 8.64)**
Type of place						
Urban	Ref	Ref	Ref			
Rural	0.44	(0.14, 1.40)	**0.36**	**(0.26, 0.49)**	0.83	(0.55, 1.27)
Condom used last time						
No	Ref	Ref	Ref			
Yes	1.13	(0.46, 2.78)	1.03	(0.73, 1.45)	0.93	(0.65, 1.32)
Previous STI						
No	Ref	Ref	Ref			
Yes	0.39	(0.12, 1.27)	1.13	(0.55, 2.31)	0.71	(0.35, 1.41)
Prior HIV testing						
No	Ref	Ref	Ref			
Yes	0.49	(0.21, 1.15)	**0.68**	**(0.47, 0.97)**	1.34	(0.81, 2.22)
Sex partners in last 12 months						
0–1	Ref	Ref	Ref			
2+	0.51	(0.13, 1.98)	0.93	(0.61, 1.42)	1.30	(0.81, 2.08)
Total lifetime sex partners						
0–1	Ref	Ref	Ref			
2+	0.99	(0.50, 1.95)	1.22	(0.97, 1.53)	1.45	(0.81, 2.57)
Proximity to National Borders (Km)						
<50 Kms	Ref	Ref	Ref			
50 Kms - 100 Kms	**3.08**	**(1.13, 8.40)**	1.15	(0.87, 1.53)	1.20	(0.69, 2.10)
100 Kms +	0.60	(0.25, 1.42)	NA	1.10	(0.70, 1.74)	
Travel time to nearest city (Hours)						
2 hrs +	Ref	Ref	Ref			
1 hr - 2 hrs	5.58	(0.80, 38.89)	1.28	(0.75, 2.19)	1.43	(0.59, 3.49)
< 1 hr	2.88	(0.63, 13.21)	1.36	(0.79, 2.34)	2.24	(0.94, 5.34)

	Zambia		Zimbabwe			
	AOR (95% CI)		AOR (95% CI)			
HIV sero-status						
HIV-	Ref		Ref			
HIV+	1.04	(0.85, 1.28)	**1.30**	**(1.01, 1.67)**		
Gender						
Male	Ref	Ref				
Female	1.30	(0.99, 1.71)	**1.37**	**(1.10, 1.69)**		
Age groups						
15–24	Ref	Ref				
25–34	**0.56**	**(0.45, 0.70)**	**0.41**	**(0.34, 0.50)**		
35–44	**0.30**	**(0.24, 0.39)**	**0.20**	**(0.15, 0.27)**		
45+	**0.19**	**(0.14, 0.26)**	**0.18**	**(0.11, 0.28)**		
Education						
Higher	Ref	Ref				
No education /Primary	0.81	(0.59, 1.11)	0.87	(0.56, 1.35)		
Secondary	0.74	(0.54, 1.01)	1.04	(0.72, 1.49)		
Wealth index combined						
Middle/Richer /Richest	Ref	Ref				
Poorest/Poorer	**0.67**	**(0.54, 0.83)**	**0.65**	**(0.50, 0.85)**		
						*(Continued on next page)*

**Table 2:** (*Continued*)

	Zambia		Zimbabwe			
	AOR (95% CI)		AOR (95% CI)			
Health insured						
No	Ref	Ref				
Yes	0.88	(0.55, 1.41)	**0.67**	**(0.45, 0.99)**		
Marital status						
Never in union	Ref	Ref				
Married/Living with a partner	**1.49**	**(1.18, 1.87)**	0.77	(0.55, 1.07)		
Divorced/Separated /Widowed	**1.71**	**(1.24, 2.35)**	**2.01**	**(1.44, 2.80)**		
Type of place						
Urban	Ref	Ref				
Rural	**0.70**	**(0.55, 0.90)**	**1.56**	**(1.16, 2.10)**		
Condom used last time						
No	Ref	Ref				
Yes	1.11	(0.90, 1.36)	0.82	(0.65, 1.02)		
Previous STI						
No	Ref	Ref				
Yes	1.18	(0.83, 1.69)	1.04	(0.62, 1.74)		
Prior HIV testing						
No	Ref	Ref				
Yes	0.94	(0.73, 1.20)	0.81	(0.60, 1.09)		
Sex partners in last 12 months						
0–1	Ref	Ref				
2+	1.09	(0.88, 1.35)	1.17	(0.90, 1.52)		
Total lifetime sex partners						
0–1	Ref	Ref				
2+	**1.24**	**(1.05, 1.46)**	**1.27**	**(1.04, 1.57)**		
Proximity to National Borders (Km)						
<50 Kms	Ref	Ref				
50 Kms - 100 Kms	**1.30**	**(1.05, 1.62)**	**0.68**	**(0.50, 0.92)**		
100 Kms +	1.17	(0.93, 1.42)	1.04	(0.82, 1.33)		
Travel time to nearest city (Hours)						
2 hrs +	Ref	Ref				
1 hr - 2 hrs	1.21	(0.94, 1.55)	1.11	(0.84, 1.47)		
< 1 hr	**1.64**	**(1.29, 2.09)**	**1.48**	**(1.08, 2.04)**		

Notes: Boldfaced numbers indicate statistical association <0.05.

Results in Table 2 are from binary logistic regression models controlled by sociodemographic variables: sex, age-group, education, wealth index combined, health insured, marital status, place type; and HIV-related information such as: Condom used last time sexual intercourse, previous STI, prior HIV testing, number of partners in last 12 months, total lifetime sex partners, proximity to national borders and travel time to nearest city.

### 3.3 Spatial distribution of mobile individuals

**Fig. 1:**
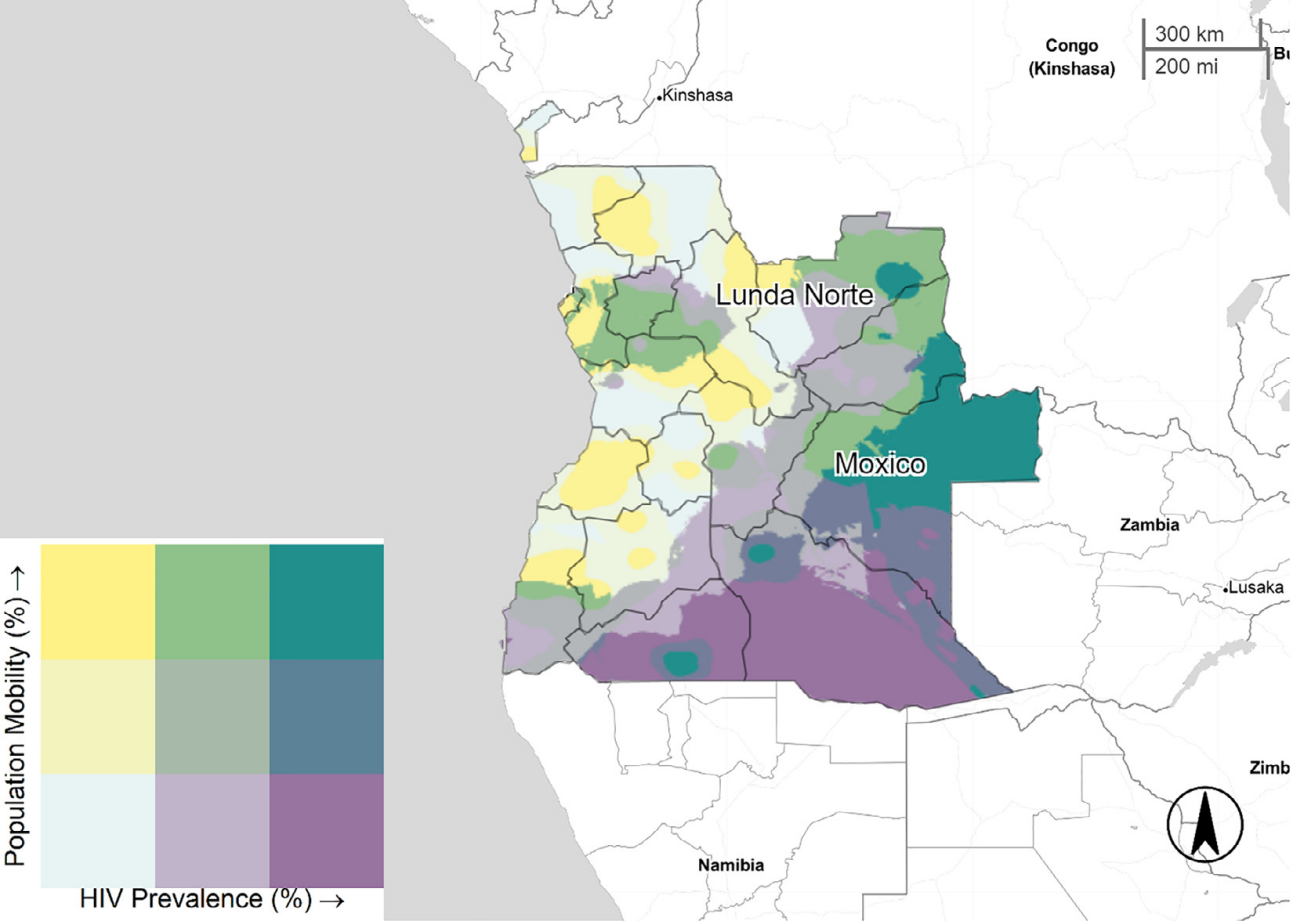
Mobile individuals versus HIV Prevalence in Angola, DHS 2015. Tertile breaks for percent of mobile individuals are 0.1% and 0.6% (range is 0.0–32.2%). Tertile breaks for HIV prevalence are 1.8% and 3.8% (range is 0.3–6.9%).

**Fig. 2:**
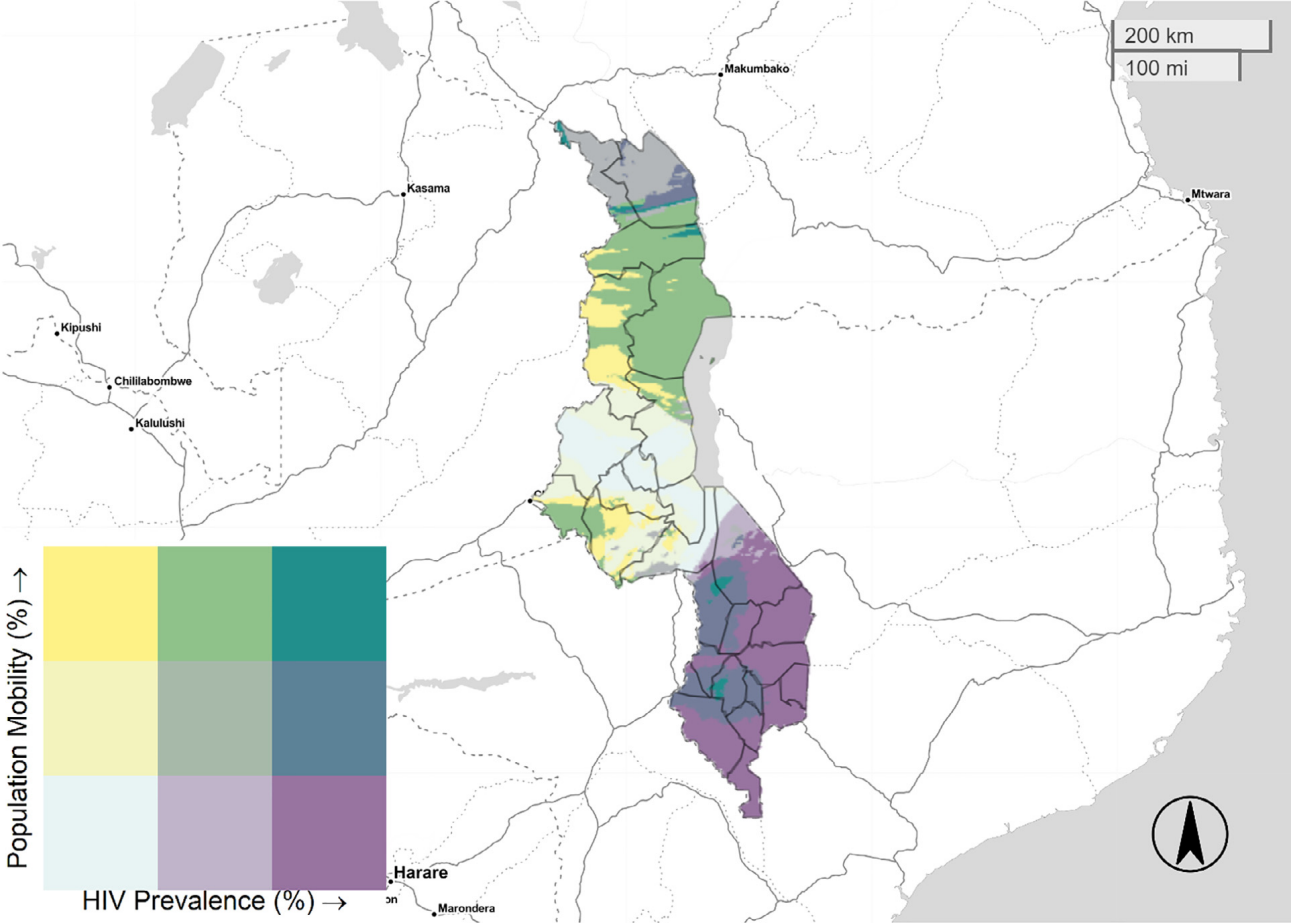
Mobile individuals versus HIV Prevalence in Malawi, DHS 2016. Tertile breaks for percent of mobile individuals are 5.4% and 7.0% (range is 2.9–10.2%). Tertile breaks for HIV prevalence are 6.1% and 7.7% (range 4.1–18%).

**Fig. 3:**
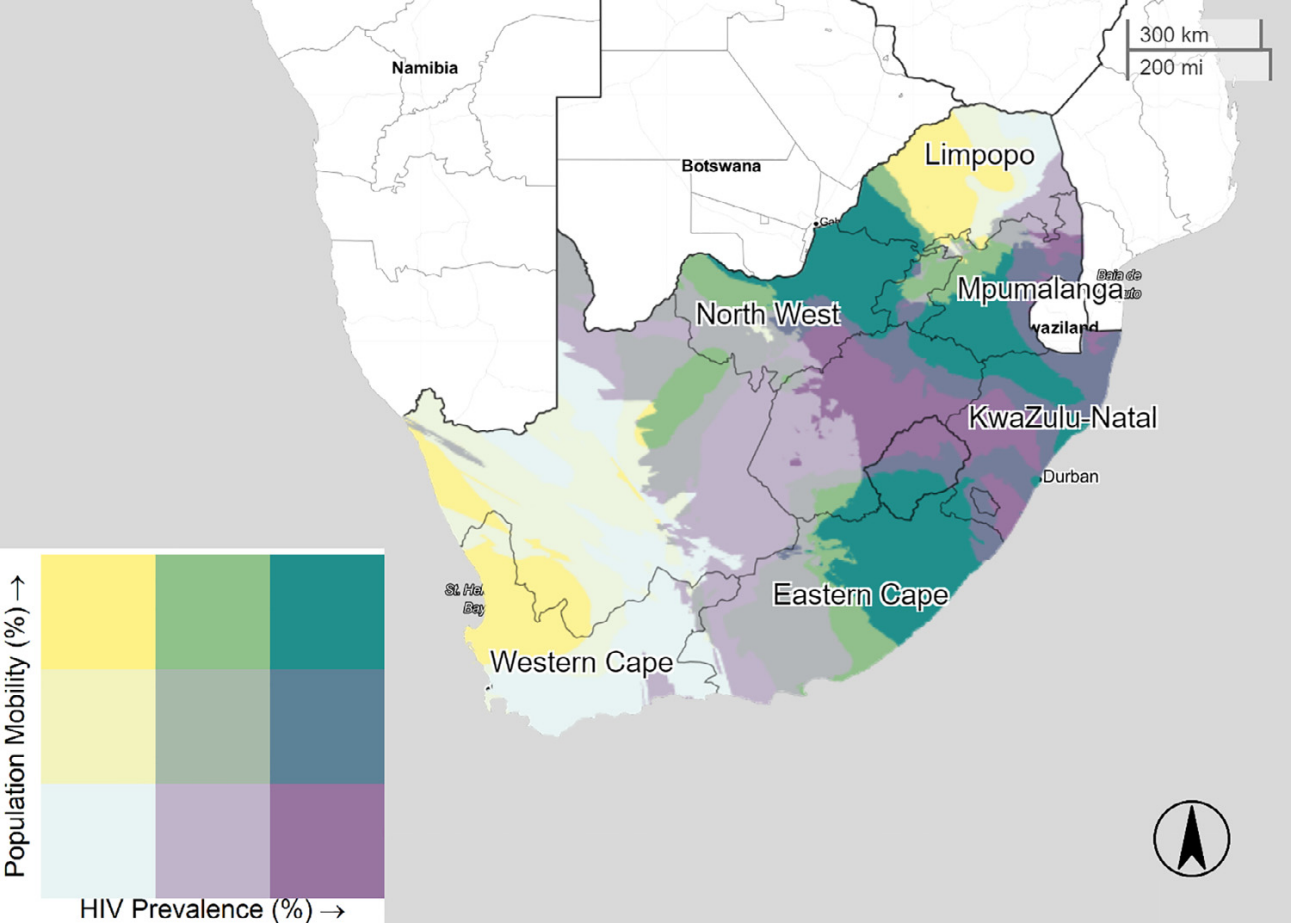
Mobile individuals versus HIV Prevalence in South Africa, DHS 2016. Tertile breaks for percent of mobile individuals are 4.1% and 5.8% (range is 0.9–18.3%). Tertile breaks for HIV prevalence are 14.8% and 19.5% (range is 7.1–33.7%).

**Fig. 4:**
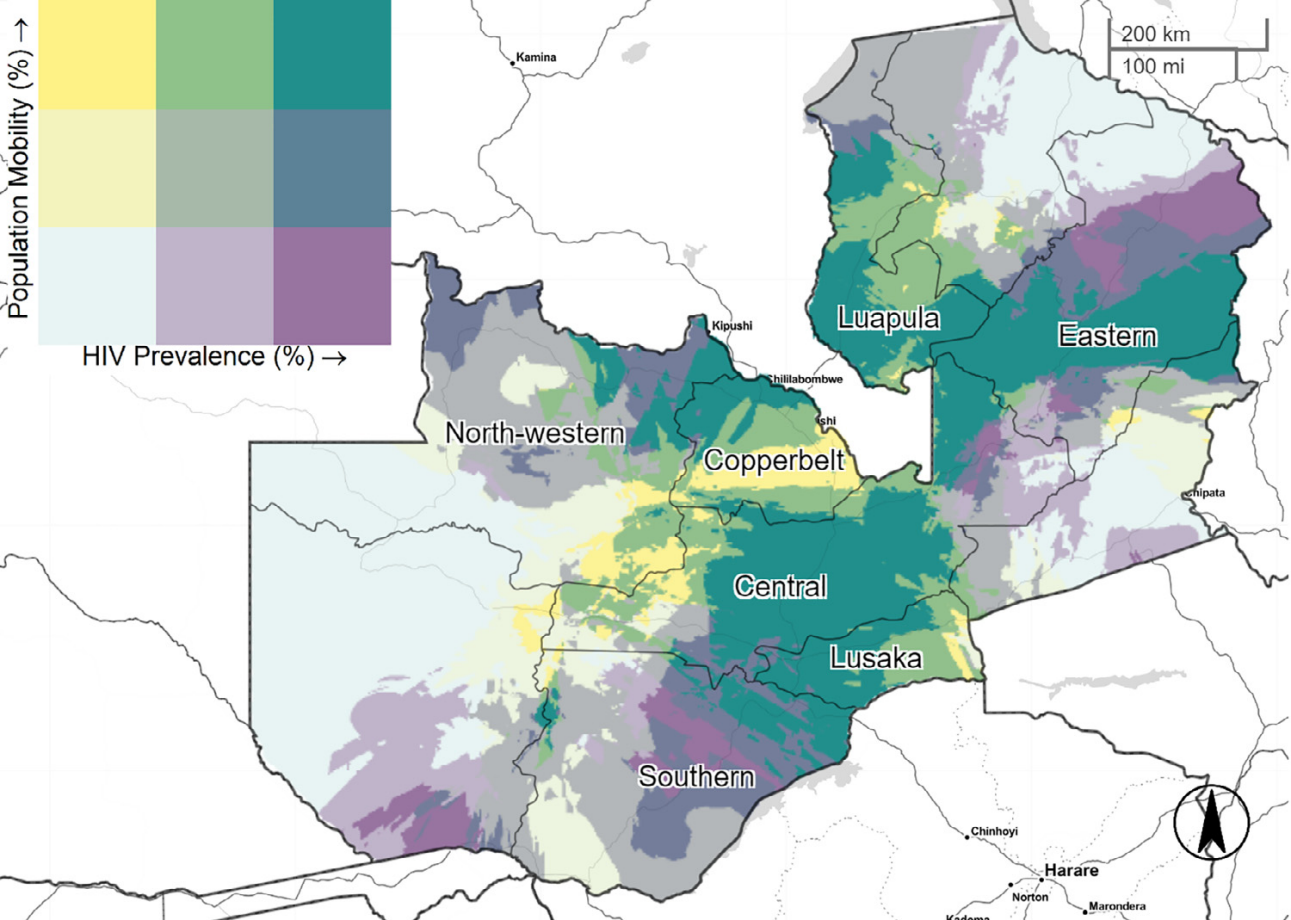
Mobile individuals versus HIV Prevalence in Zambia, DHS 2013–2014. Breaks for percent of mobile individuals are 7.7% and 8.6% (range is 5.6–11.2%). Breaks for HIV prevalence are 9.9% and 10.7% (range is 7.8–16.1%).

**Fig. 5:**
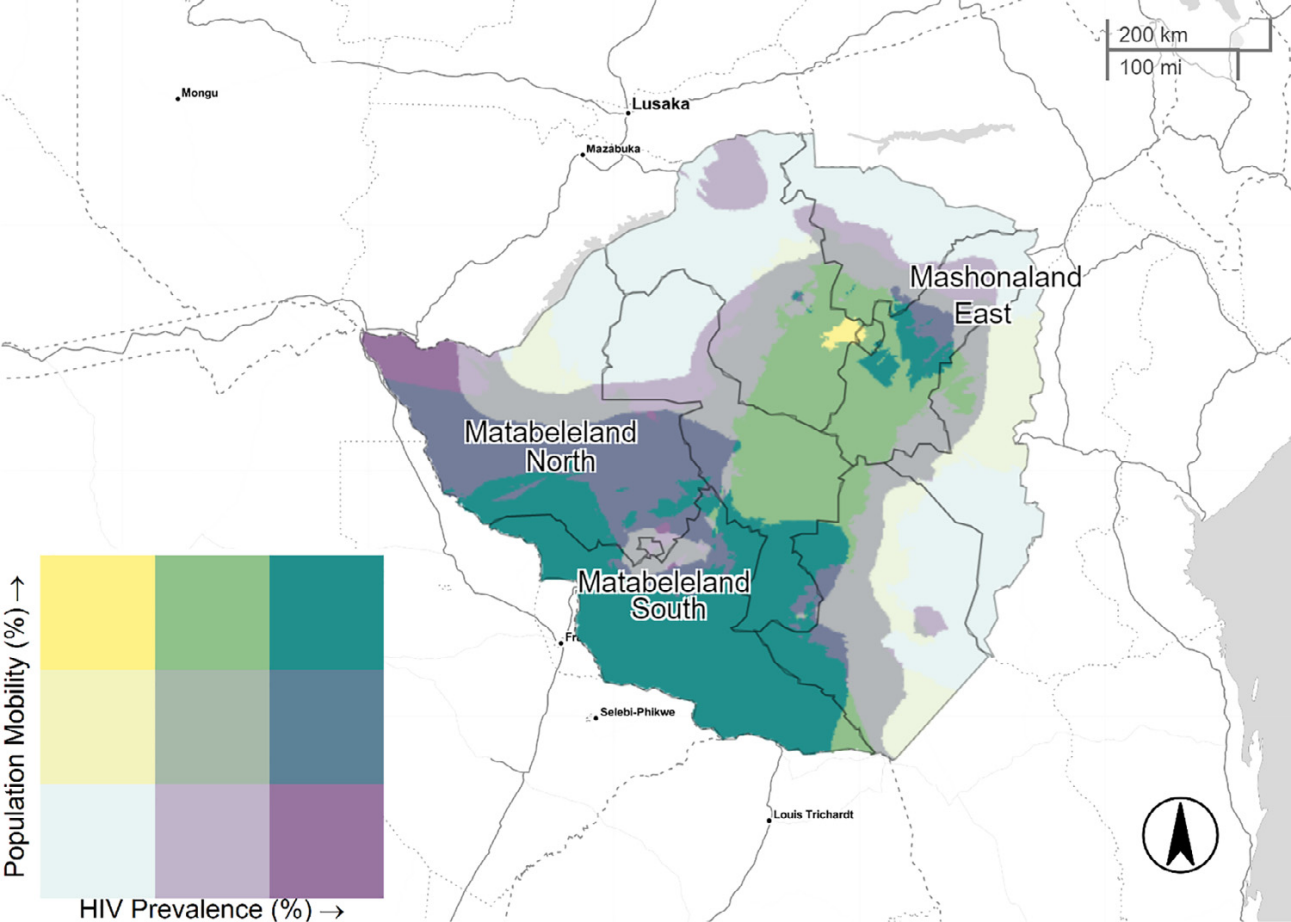
Mobile individuals versus HIV Prevalence in Zimbabwe, DHS 2015. Tertile breaks for percent of mobile individuals are 6.9% and 8.8% (range is 4.3–12.6%). Tertile breaks for HIV prevalence are 12.6% and 16.3% (range is 7.8–25.5%).

Figs. 1–5 illustrate the spatial distribution of mobile individuals and HIV prevalence for all countries. We observed a spatial link between the two factors in Angola, South Africa, Zambia, and Zimbabwe. High mobility-high HIV prevalence areas were present at national borders in these four countries. In Angola, mobile individuals were located at the east border, distributed between Lunda Norte and Moxico provinces with an HIV prevalence ranging from 3.8% to 6.9% (Fig. 1). In South Africa, five provinces: North-West, Limpopo, Mpumalanga, KwaZulu-Natal and Eastern Cape showed strong presence of mobile individuals in areas with high HIV prevalence (≥19.5%), whereas Western Cape showed only consistent presence of mobile individuals near to Cape Town (Fig. 3). Mobile individuals in high HIV burden areas in Zambia (≥10.7%) were located in Central, Lusaka, Muchinga, and borders of Copperbelt, Luapula, North-western, and Southern provinces (Fig. 4). Finally, Zimbabwe's provinces of Mashonaland East, Matabeleland North and South (Fig. 5) exhibited high density of mobile individuals in areas with high HIV prevalence (≥16.3%).

## 4 Discussion

This study provides a national-level characterization of health and socioeconomic determinants of mobile individuals across five different HIV context in Southern Africa. We found that younger individuals were more likely to migrate in all countries. In addition, females, and individuals who divorced, separated, or widowed were more likely to be mobile and have lived in the place of residence for less than one year. Neither education nor wealth index combined were associated with mobility. Moreover, we identified a significant association between HIV serostatus and mobility only in Zimbabwe. Conversely, there were no significant differences in sexual behavior covariates by mobility status except for the total lifetime sexual partners. Finally, our spatial distribution agrees with our multivariate analysis covariates reported, showing high density of mobile population in areas with high HIV prevalence in Zimbabwe.

Our findings showed that age was the most consistent factor linked to population movement in all five countries. Mobile individuals were about five years younger compared to non-mobile individuals. This result is consistent with global statistics on population mobility where the majority of mobile individuals are young and middle-aged people (R Core Team 2016). Prior research has documented how weak family ties, ambition, and livelihood prospects might encourage young individuals to move to urban settings (Sandefur and Scott, 1981). In an HIV context, the International Organization for Migration (IOM) identified mobile individuals and labor migrants as key populations for HIV epidemics in southern Africa (Migration IOo 2010). Although young populations are not a risk factor themselves, conditions and lifestyles within mobility process might foster risky behaviors, increasing their vulnerability to HIV. For marital status, other studies have found the effect of marriage on migration-related causes (Anglewicz and Anglewicz, 2012; Coffee et al., 2005; Reniers, 2003). Long partner separation, lack of partner vigilance and concurrent relationships due to job mobility are high-risk factors exposing middle aged individuals to HIV. We found increased odds of having two or more lifetime sexual partners in Zambia (24%) and Zimbabwe (27%), respectively.

Although men have traditionally outnumbered women in mobile populations in Africa, female migrants are more rapidly increasing than male migrants (R Core Team 2016). We found that Zimbabwean women were more likely to move than men. In recent years, reasons including economic prospects and availability of undesired low-skill occupations have influenced women to move more in southern Africa (Migration IOf 2019). Among labor-related mobile individuals, 33.7% are females in Zimbabwe (Migration IOf 2018). Social environment such as pressure for child marriages (termed as *bridewealth*) might motivate women to move in Zimbabwe (Migration IOf 2018). In an HIV context, the role between mobile individuals and gender has been extensively documented in female workers (Lurie et al., 2003; Camlin et al., 2014). An increased HIV prevalence was observed in females who slept away from home than in males (Migration IOf 2019). Additionally, Brown et al. (Brown et al., 2018) reported that young females are less likely to know their HIV status or where and when they acquired HIV in several African countries. Because females possess these critical livelihood and work characteristics that impact their vulnerability to HIV, females, particularly mobile females, should be a priority population for health interventions in Zambia and Zimbabwe.

This study also highlights increased mobility in border provinces and areas with high HIV prevalence. Although informal travel and trade are common traits of Africa's socioeconomic landscape, transporters and border trade communities have been linked to high HIV prevalence (Carswell et al., 1989). Strong spatial mobility patterns were observed in Angola, Zambia, South Africa, and Zimbabwe except for Malawi. Angola has low overall HIV prevalence (2%) and subtle variations (>4%) in south east provinces (Instituto Nacional de Estatística - INE/Angola 2017). With the growth of its economy, Angola has attracted and received individuals from its neighbors in recent years (Migration AOo). Its economic dynamics might explain the high mobility-high HIV prevalence pattern in Moxico region, which shares border corridors with the Democratic Republic of Congo and Zambia. It is worth nothing the high mobile pattern of individuals near to Luanda, a region with historically low HIV prevalence (<2%).

As the main destination of Southern Africa Development Community (SACD) for mobile populations, South Africa's economy has been determined by agricultural and mining activities. In an HIV context, sexual networking that arises in response to these working activities, has been recognized to contribute to the HIV spread (Networks HaD 2005). In the north, individuals looking for opportunities in mining areas (e.g., Rustenburg and Steelpoort) might explain the substantial concentration of highly mobile individuals. The provinces of North West and Limpopo share multiple border posts with Botswana (Groblersbrug, Ramatlabama, and Skilpadshek) and Zimbabwe (Beitbridge). Lurie et al. has previously reported high rates of HIV in labor migrants of Carletonville's goldmines (Lurie et al., 2003). Also, cross-border interaction with less contained HIV countries (Swaziland and Lesotho) combined with the propensity of mobile individuals to settle close to highly urbanized areas might explain the high mobility-high HIV pattern of Eastern Cape, Mpumalanga and KwaZulu Natal. About fifty-percent of Lesotho males work in South Africa (Banati, 2007). Of interest is the high mobility pattern close to Cape Town, a region with an HIV prevalence rate of 17.1% (National Department of Health 2019). These documented interactions between labor-sending and labor-receiving regions and good transport infrastructure might explain high-mobility patterns in South Africa (Lurie et al., 2003; Networks HaD 2005; Banati, 2007). In Zambia, the reasons for the increased mobility areas could be numerous. First, we note that Zambia shares borders with eight countries, making it a common corridor for the Southern African Development Community (International Organization for Migration, 2006). Second, Zambia is considered as a peaceful and politically stable country, making it a place of origin, transit and destination for numerous types of mobile populations including refugees from its neighbors (Migration IOf 2019). Moreover, Zambia's mobile individuals are predominantly urban-to-urban population which can explain the high-mobility pattern in and out of Lusaka province (20). We also noted that Chirundu has the largest commercial sex industry in the country (USAID 2004). In regard to Zimbabwe, high mobility areas in the Matabeleland provinces might be explained by irregular nationals who have been prosecuted, and later deported through the Plumtree border Post (Botswana) and the Beitbridge Border Post (South Africa) (Galvin, 2015). Those border points are important travel routes that economically integrate Bulawayo with Francistown and Gwanda with Messina and Johannesburg (Moyo, 2017). However, the rates of HIV serostatuses for this population group is not quantified in the literature. In Zimbabwe, mobile individuals reported 48% higher odds of living near main cities.

Our study has several limitations. First, due to the lack of characterization related to the change of residence in the survey, we defined mobility status based only on years lived in residence, which might not be as accurate to track and characterize population mobility. No information on trip motivation, traveled distance, duration and frequency might lead to some underestimated results of the real burden of population mobility as previous research suggests (Camlin and Charlebois, 2019). Furthermore, to our knowledge data regarding the HIV serostatus of individuals that move across Southern corridors, specifically in the studied countries are not available. From the DHS datasets perspective, a selection bias may be present in the sample since only selected individuals who were willing to participate in the survey and meet the inclusion criteria were used for the estimates. This could lead to spurious associations with human mobility and mapping errors for HIV prevalence in some countries. Hence, our results should be interpreted with caution. Also, the DHS does not measure viral load suppression (VLS) biomarker, which is critical in infectious determination and treatment success for HIV positive individuals. Both these factors are essential in the determination process towards the 90–90–90 targets (Cuadros et al., 2019). Finally, because DHS datasets are cross-sectional surveys, our study was limited in deriving conclusions about the direction of the causality between population mobility and HIV serostatus.

## 5 Conclusions

This study is one of the first studies exploring health and social determinants and HIV serostatus on mobile individuals at national level, and the use of spatial approaches to investigate geographical structures of these populations across different HIV epidemic intensities in Southern Africa. Different implications might be derived from this study. First, it is essential that authorities identify and remove barriers of health services where mobile individuals are most likely to be found. For the gendered results, countries might focus on sexual education and condom usage for females. Additional efforts might focus on scaling up testing interventions to assess whether HIV acquisition occurs premigration or postmigration in young females. Also, public health systems can facilitate treatment for HIV-positive individuals who move from one location to another, increasing their adherence and accessibility to HIV services. Second, spatial patterns of high mobility-high HIV prevalence at country borders evidence the needs of healthcare systems with HIV serostatus data for mobile individuals in geographically contiguous countries for surveillance and attention. Therefore, regional initiatives such as Corridors of Hope might be re-implemented and extended to facilitate sexual health services (USAID 2004), ensuring that these marginal groups are not left behind in the context of HIV and the UNAIDS 90–90–90 targets. Also, HIV educational strategies might be available in different languages to diminish knowledge gaps. Third, analysis such as the one we have generated should give policymakers a more precise geographic focus to enhance their impact, especially in potential areas identified for high mobility and high HIV prevalence. These areas can benefit from resource allocation for free effective HIV interventions such as antiretroviral therapy (ART), voluntary medical male circumcision (VMMC), and oral pre-exposure prophylaxis (PrEP). To achieve epidemic control in terms of the UNAIDS 90–90–90 strategy for marginal groups, future research needs to focus on understanding the causes and consequences of health disparities for mobile populations at different scales.

## Author's contribution

ECA carried out study implementation, analysis and interpretation of data and major contribution to writing. HYK, GNM, ZM, AA carried out major contribution to writing. DFC carried out study design, analysis and interpretation of data and major contribution to writing. All authors read and approved the final manuscript.

## Declaration of Competing Interest

None.

## Appendix Supplementary materials

Image, application 1
