# Supplementary material for: Associated health and social determinants of mobile populations across HIV epidemic gradients in Southern Africa
Source: J Migr Health. 2021 Mar 26;3:100038. doi: 10.1016/j.jmh.2021.100038 (PMC8352162; doi:10.1016/j.jmh.2021.100038)
Supplement: Supplementary file 1 [file mmc1.pdf]

SUPLEMENTARY MATERIALS FOR:

ASSOCIATED HEALTH AND SOCIAL DETERMINANTS OF MOBILE POPULATIONS ACROSS HIV EPIDEMIC GRADIENTS IN SOUTHERN AFRICA

Esteban Correa-Agudelo[1,2], Hae-Young Kim[3,4,5], Godfrey N. Musuka[6], Zindoga Mukandavire[7,8], Adam Akullian[9,10], Diego F. Cuadros[1,2*]

[1]*Department of Geography and Geographic Information Science, University of Cincinnati, Cincinnati, USA*

[2]*Health Geography and Disease Modeling Laboratory, University of Cincinnati, Cincinnati, USA*

[3]*Africa Health Research Institute, KwaZulu-Natal, South Africa*

[4]*KwaZulu-Natal Research Innovation and Sequencing Platform (KRISP) KwaZulu-Natal, South Africa*

[5]*Department of Population Health, New York University Grossman School of Medicine*

[6]*ICAP at Columbia University, Harare, Zimbabwe*

[7]*Centre for Data Science, Coventry University, UK*

[8]*School of Computing, Electronics and Mathematics, Coventry University, UK*

[9]*Institute for Disease Modeling, Global Good Fund, Bellevue, Washington, USA*

[10]*Department of Global Health, University of Washington, Seattle, Washington, USA*

** Reprints or correspondence: Diego F. Cuadros, PhD, E-mail: diego.cuadros@uc.edu. Department of Geography and Geographic Information Science, University of Cincinnati, Cincinnati, OH, 45221. Telephone: (513) 556-3423. Fax: (513) 556-3370.*

APPENDIX A: Covariate selection criteria and definitions

All covariates were selected according to an evidence synthesis process of relevant references (1-19). This section describes the association of migrancy with sociodemographic cofactors, HIV related and geospatial covariates. First, we make use of a simplified evidence synthesis protocol to select covariates of interest to migration and HIV. Then, an implied graph (IG) was built to infer causal effects to the observational data. Finally, backdoor criterion was performed to remove open paths, check for colliders and overcontrol in the implied graph. The result is the following directed acyclic graph (DAG) for this hypothetical study:

Figure A.1. Hypothetical DAG for the study

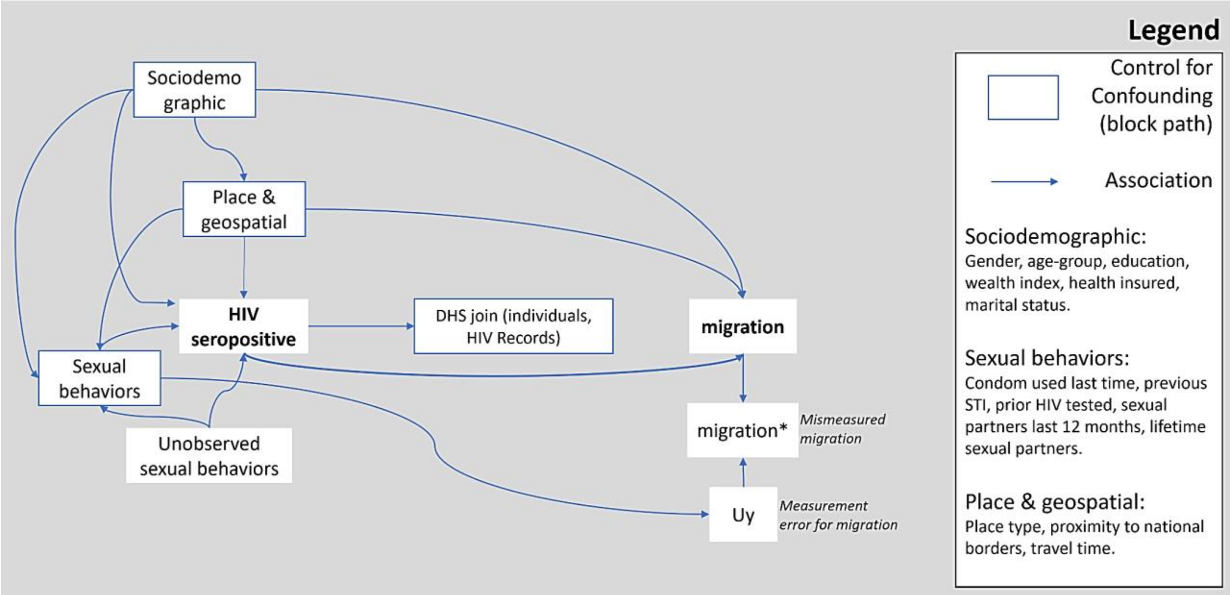

All covariates are readily available in the DHS surveys and are described as follows:

Age has mixed effects in migration and HIV exposure. For example, migrant population have been predominantly young (2, 20), whereas early age population have higher odds of sexual debut which is associated with numerous other risk factors such as HIV acquisition (18).

Question V013 "*Age in 5-year groups*" was grouped in ten-year groups (15-24, 25-34, 35-44, and 45+).

Education, wealth index combined, and health insured are key covariates to characterize migrant population. Three categories (no education or primary, secondary, and higher education) were derived from question V106 "*highest educational level*". Wealth index (V190) has been group into poorer/poorest and middle/richer/richest. V481 "*covered by health insurance*" is also included.

Marital status represents a structural factor that is close to migration and HIV exposure (12, 21). The information was determined from question V501 "*current marital status*" and categorized as: never in union, married/living with a partner, and divorced/separated/widowed. Prior research has shown how migration differs between urban and rural settings (10, 22). Question V102 "*type of place of residence*" was included for this study.

Prior HIV tested and previous STI are well known entry points of HIV transmission and treatment (14). DHS question V781 "*Ever been tested for HIV*", and V763A "*had any STI in last 12 months*" were used with appropriate levels (yes, no). An individual's number of sexual partners correlates with sexual activity and HIV exposure27. Surveys ask men and women their number of partners in the past year, and lifetime. We used these responses to construct a picture of sexual relationships in the short and long term for migrant population. Questions V766b "*Number of sex partners, including spouse, in last 12 months*", and V836 "*Total lifetime number of sex*" where used to derive the information and grouped into two categories: 0–1 sex partner and 2+ partners.

Proximity to national borders and travel time have been identified as factors associated with the risk of HIV infection linked to mobile individuals (13). We used DHS covariates *"Proximity to national borders and travel time"* as geospatial covariates of migration (23). Proximity to national borders was grouped into three categories: *<50 km* category to track people who live in areas closer than 50 km to national borders; 50 km-100km; and *100km+* which included those residents who live in areas farther than 100 km distance. Travel time was grouped as follows: *<1 hr.* category to track people who live in areas were the travel time to nearest city is less than 60 minutes; *1 hr-2 hrs*; and *2 hrs+* which included those residents who live in areas with a travel time higher than 120 minutes. As an example, Figure A.2. shows a schematic representation of the inclusion criteria workflow for the outcome and exposures of Zambia's male dataset. First, duplicated IDs are removed. Then, males with non-HIV valid information and non-mobile status data are excluded. Lastly, Zambian males with unknown sexual covariates are omitted in the final sample.

Figure A.2. Schematic representation of the inclusion criteria workflow for Zambia's 2018 male recode.

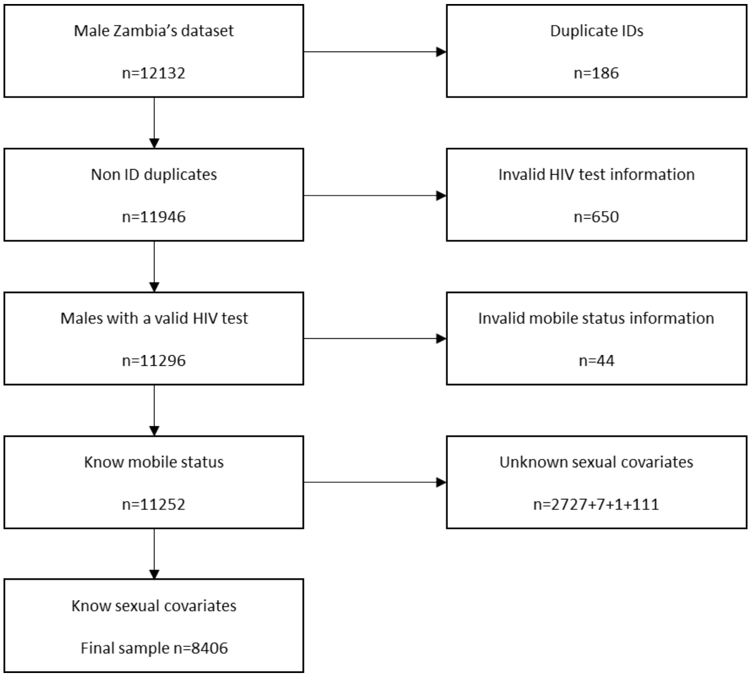

APPENDIX B: Statistical Analysis

Complementarily to the evidence synthesis and DAG analysis, we performed a Variance Inflated Factor (VIF) stepwise model selection to account for multicollinearity. Selected variables were evaluated using the *jtools* package on R to account for the stratified two-stage sampling in each country. VIF stepwise model selection used the correlation matrix computed over the samples, and measure collinearity using a multiple regression, and taking the ratios of all given models divided by the variance of the single coefficients for each variable if it were fitted individually. Finally, all variables less than five (5.00) VIF score were allowed to be included in the final adjusted model. For our specific DAG, any variable showed high correlation among the dataset and all adjusted paths and individual attributable factors for migration were included in the final model. Noteworthy is the case of Angola VIF scores. This could be explained by the relatively small proportion of migrants in this country (1%) which affects the results. R programming environment (24) and *survey* (25) package were used to fit models.

Table B1. Variance inflated factor (VIF) model selection results

	**Angola**	**Malawi**	**South Africa**	**Zambia**	**Zimbabwe**
	VIF Score	VIF Score	VIF Score	VIF Score	VIF Score
HIV sero-status					
HIV-	Ref	Ref	Ref	Ref	Ref
HIV+	2.15	1.48	1.64	1.54	1.32
Gender					
Male	Ref	Ref	Ref	Ref	Ref
Female	5.77	1.81	1.94	2.18	2.02
Age groups					
15-24	Ref	Ref	Ref	Ref	Ref
25-34	11.79	2.85	4.17	2.52	2.00
35-44	11.79	2.85	4.17	2.52	2.00
45+	11.79	2.85	4.17	2.52	2.00
Education					
Higher	Ref	Ref	Ref	Ref	Ref
No education/Primary	4.05	3.14	1.78	1.80	1.84
Secondary	4.05	3.14	1.78	1.80	1.84

Wealth index combined					
Middle/Richer/Richest	Ref	Ref	Ref	Ref	Ref
Poorest/Poorer	3.22	1.53	2.89	1.61	1.56
Health insured					
No	Ref	Ref	Ref	Ref	Ref
Yes	4.03	1.52	1.27	1.17	1.55
Marital status					
Never in union	Ref	Ref	Ref	Ref	Ref
Married/Living with a partner	5.02	4.16	2.79	2.09	3.09
Divorced / Separated / Widowed	5.02	4.16	2.79	2.09	3.09
Place type					
Urban	Ref	Ref	Ref	Ref	Ref
Rural	5.03	1.97	2.03	2.14	2.29
Condom used last time					
No	Ref	Ref	Ref	Ref	Ref
Yes	3.04	1.75	1.68	1.27	1.91
Previous STI					
No	Ref	Ref	Ref	Ref	Ref
Yes	2.22	1.51	1.16	1.14	1.48
Prior HIV testing					
No	Ref	Ref	Ref	Ref	Ref
Yes	3.42	1.73	1.26	1.35	1.63
Sex partners in last 12 months					
0-1	Ref	Ref	Ref	Ref	Ref
2+	2.22	1.44	1.27	1.24	1.32
Total lifetime sex partners					
0-1	Ref	Ref	Ref	Ref	Ref
2+	1.60	1.52	2.11	1.75	1.56
Proximity to National Borders (Km)					
<50 Kms	Ref	Ref	Ref	Ref	Ref
50 Kms - 100 Kms	9.29	1.37	1.93	1.36	1.56
100 Kms +	9.29	NA	1.93	1.36	1.56
Travel time to nearest city (Hours)					
< 1 hr	Ref	Ref	Ref	Ref	Ref
1 hr - 2 hrs	13.16	1.67	1.78	1.55	2.13
2 hrs +	13.16	1.67	1.78	1.55	2.13

Table B2. Unadjusted odds ratios showing associations between migration and control variables in Angola, Malawi, South Africa, Zambia, and Zimbabwe

	**Angola**	**Malawi**	**South Africa**
	OR (95% CI)	OR (95% CI)	OR (95% CI)
HIV sero-status			
HIV-	Ref	Ref	Ref

HIV+	0.86	(0.20 , 3.63)	1.16	(0.84 , 1.61)	1.37	(0.88 , 2.15)
Gender						
Male	Ref		Ref		Ref	
Female	0.50	(0.24 , 1.05)	1.36	(1.06 , 1.73)	1.24	(0.87 , 1.77)
Age groups						
15-24	Ref		Ref		Ref	
25-34	**0.41**	**(0.22 , 0.76)**	**0.64**	**(0.52 , 0.80)**	0.97	(0.61 , 1.54)
35-44	**0.08**	**(0.03 , 0.22)**	**0.30**	**(0.21 , 0.44)**	**0.54**	**(0.31 , 0.94)**
45+	**0.01**	**(0.0 , 0.08)**	**0.19**	**(0.10 , 0.34)**	**0.15**	**(0.06 , 0.35)**
Education						
Higher	Ref		Ref		Ref	
No education/Primary	2.64	(0.64 , 10.92)	**0.35**	**(0.21 , 0.58)**	1.63	(0.77 , 3.47)
Secondary	2.16	(0.54 , 8.70)	0.81	(0.48 , 1.37)	1.40	(0.72 , 2.73)
Wealth index combined						
Middle/Richer/Richest	Ref		Ref		Ref	
Poorest/Poorer	0.84	(0.27 , 2.68)	**0.68**	**(0.51 , 0.89)**	1.64	1.09 , 2.47)
Health insured						
No	Ref		Ref		Ref	
Yes	1.03	(0.26 , 4.02)	**2.58**	**(1.52 , 4.38)**	**0.36**	**(0.20 , 0.64)**
Marital status						
Never in union	Ref		Ref		Ref	
Married/Living with a partner	**0.48**	**(0.25 , 0.95)**	0.83	(0.61 , 1.12)	1.26	(0.83 , 1.90)
Divorced / Separated / Widowed	0.99	(0.39 , 2.52)	1.16	(0.71 , 1.89)	2.0	(0.93 , 4.30)
Type of place						
Urban	Ref		Ref		Ref	
Rural	0.65	(0.14 , 3.04)	**0.28**	**(0.21 , 0.37)**	0.84	(0.58 , 1.22)
Condom used last time						
No	Ref		Ref		Ref	
Yes	1.74	(0.80 , 3.79)	1.20	(0.92 , 1.58)	1.00	(0.71 , 1.42)
Previous STI						
No	Ref		Ref		Ref	
Yes	0.34	(0.11 , 1.12)	1.26	(0.66 , 2.40)	0.79	(0.39 , 1.58)
Prior HIV testing						
No	Ref		Ref		Ref	
Yes	**0.42**	**(0.19 , 0.93)**	**0.94**	**(0.68 , 1.29)**	1.52	(0.92 , 2.51)
Sex partners in last 12 months						
0-1	Ref		Ref		Ref	
2+	0.72	(0.21 , 2.39)	0.91	(0.63 , 1.31)	1.30	(0.83 , 2.04)
Total lifetime sex partners						
0-1	Ref		Ref		Ref	
2+	1.07	(0.53 , 2.19)	1.06	(0.87 , 1.29)	1.29	(0.78 , 2.12)
Proximity to National Borders (Km)						
<50 Kms	Ref		Ref		Ref	
50 Kms - 100 Kms	2.57	(0.51 , 12.92)	**1.75**	**(1.30 , 2.34)**	1.17	(0.69 , 1.98)
100 Kms +	0.58	(0.31 , 1.08)	NA		1.23	(0.77 , 1.95)
Travel time to nearest city (Hours)						

2 hrs +	Ref	Ref	Ref
1 hr - 2 hrs	**6.70 (1.06 , 42.22)**	1.37 (0.80 , 2.36)	1.48 (0.66 , 3.34)
< 1 hr	**3.41 (1.41 , 8.25)**	**2.24 (1.32 , 3.80)**	2.04 (0.94 , 4.43)

	**Zambia**	**Zimbabwe**
	OR (95% CI)	OR (95% CI)
HIV sero-status		
HIV-	Ref	Ref
HIV+	1.15 (0.95 , 1.38)	**1.05 (0.84 , 1.31)**
Gender		
Male	Ref	Ref
Female	**1.42 (1.12 , 1.81)**	**1.28 (1.09 , 1.51)**
Age groups		
15-24	Ref	Ref
25-34	**0.75 (0.62 , 0.89)**	**0.41 (0.34 , 0.49)**
35-44	**0.41 (0.33 , 0.51)**	**0.20 (0.16 , 0.26)**
45+	**0.23 (0.18 , 0.30)**	**0.17 (0.11 , 0.26)**
Education		
Higher	Ref	Ref
No education/Primary	**0.57 (0.44 , 0.72)**	1.37 (0.96 , 1.97)
Secondary	0.82 (0.64 , 1.06)	**1.78 (1.31 , 2.42)**
Wealth index combined		
Middle/Richer/Richest	Ref	Ref
Poorest/Poorer	**0.45 (0.38 , 0.53)**	**0.70 (0.55 , 0.88)**
Health insured		
No	Ref	Ref
Yes	1.15 (0.77 , 1.72)	**0.53 (0.38 , 0.74)**
Marital status		
Never in union	Ref	Ref
Married/Living with a partner	**0.73 (0.61 , 0.89)**	**0.44 (0.34 , 0.56)**
Divorced / Separated / Widowed	1.05 (0.77 , 1.43)	1.29 (0.99 , 1.68)
Type of place		
Urban	Ref	Ref
Rural	**0.45 (0.38 , 0.54)**	1.05 (0.83 , 1.33)
Condom used last time		
No	Ref	Ref
Yes	**1.31 (1.05 , 1.64)**	**1.44 (1.20 , 1.73)**
Previous STI		
No	Ref	Ref
Yes	1.28 (0.94 , 1.75)	1.20 (0.74 , 1.95)
Prior HIV testing		
No	Ref	Ref
Yes	1.15 (0.93 , 1.43)	0.79 (0.61 , 1.04)
Sex partners in last 12 months		
0-1	Ref	Ref

2+	0.96	(0.78	,	1.18)	**1.33**	**(1.05**	**,**	**1.69)**
Total lifetime sex partners								
0-1		Ref				Ref		
2+	0.99	(0.83	,	1.19)	1.08	(0.91	,	1.28)
Proximity to National Borders (Km)								
<50 Kms		Ref				Ref		
50 Kms - 100 Kms	**1.44**	**(1.14**	**,**	**1.82)**	**0.71**	**(0.52**	**,**	**0.98)**
100 Kms +	0.95	(0.75	,	1.20)	1.13	(0.88	**,**	1.45)
Travel time to nearest city (Hours)								
2 hrs +		Ref				Ref		
1 hr - 2 hrs	1.22	(0.95	,	1.54)	1.16	(0.86	,	1.56)
< 1 hr	**2.48**	**(1.99**	**,**	**3.08)**	**1.44**	**(1.10**	**,**	**1.88)**

Notes: Boldfaced numbers indicate statistical association <0.05.
Results in Table 3 are from binary unadjusted logistic regression models by sociodemographic variables: sex, age-group, education, wealth index combined, health insured, marital status, place type; and HIV-related information such as: Condom used last time sexual intercourse, previous STI, prior HIV testing, number of partners in last 12 months, total lifetime sex partners, proximity to national borders and travel time to nearest city.

APPENDIX C: Continuous surfaces and bivariate maps

Disease mapping is the process of visually depicting geographically indexed data in a spatial referenced distribution for explanatory purposes. Interpolation methods are commonly used to predict unknown values for any geographic data point. Classical methods such as Inverse Distance Weighting (IDW) interpolate the value at any location as a function of the distance to known values at observed locations. In other words, nearby points are weighted much higher than remote ones (26). Kriging is similar to IDW in that it weights the surrounding observed values to make a reliable prediction (27). However, Kriging also includes the spatial arrangement of points by determining the spatial autocorrelation (how correlated variables vary along distances) through a semi variogram calculation, accounting for data closeness, spatial continuity and redundancy. Consider the case of estimating the migration proportion at some unsampled location in Zimbabwe (See Figure C3.1):

Figure C3.1. Sampling locations for Zimbabwe (PSU)

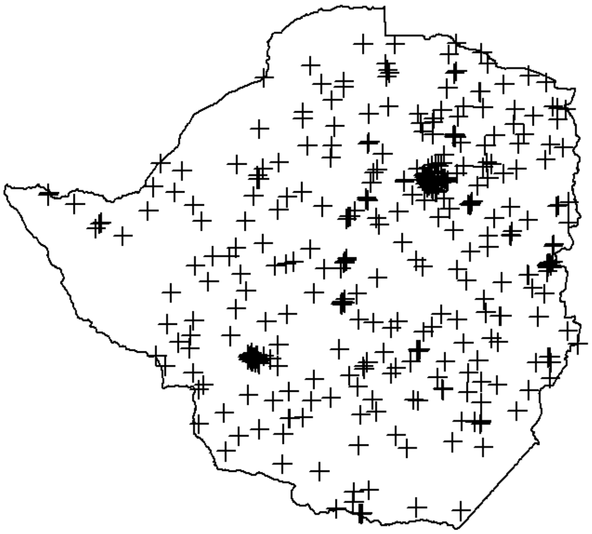

Now, consider the equation of a best linear unbiased predictor kriging and its covariance:

$$y^*(u_0) = \sum_{\alpha=1}^{n} w_\alpha \, y(u_\alpha)$$

$$\sigma_\epsilon^2(u) = C(0) - \sum_{\alpha=1}^{n} w_\alpha \, C(u - u_\alpha)$$

The goal is to take and decompose the total variability of a spatial system in two components. One side we have the variable we want to model (migration, or HIV prevalence), and it is considered known *(u_α)*. On the other side, we would have the residual component or the error of that interpolation. Residual is considered not known. Weights are unbiased and minimize the estimation variance. Then, the idea is to find the weights that minimize the variance (See Figure AC.2). The process of adapting variability, or heterogeneity, in the unknown process is of particular importance in spatial datasets. Therefore, the K-Bessel (also known as the Matern kernel) model has been used; this has become popular in the spatial statistics literature for gaussian processes (27). This model tends to produce surfaces that are on a very fine scale smoother than some other models (such as the exponential or spherical). For the range search, a fixed value of 50 km distance has been used for all countries. Each variogram has been tuned following that range. Then, a map is created joining estimated surfaces of two variables into a bivariate map.

Figure C3.2. Known locations (PSU) and kriging variance. Angola (a); Malawi (b); South Africa (c); Zambia (d); and Zimbabwe (e)

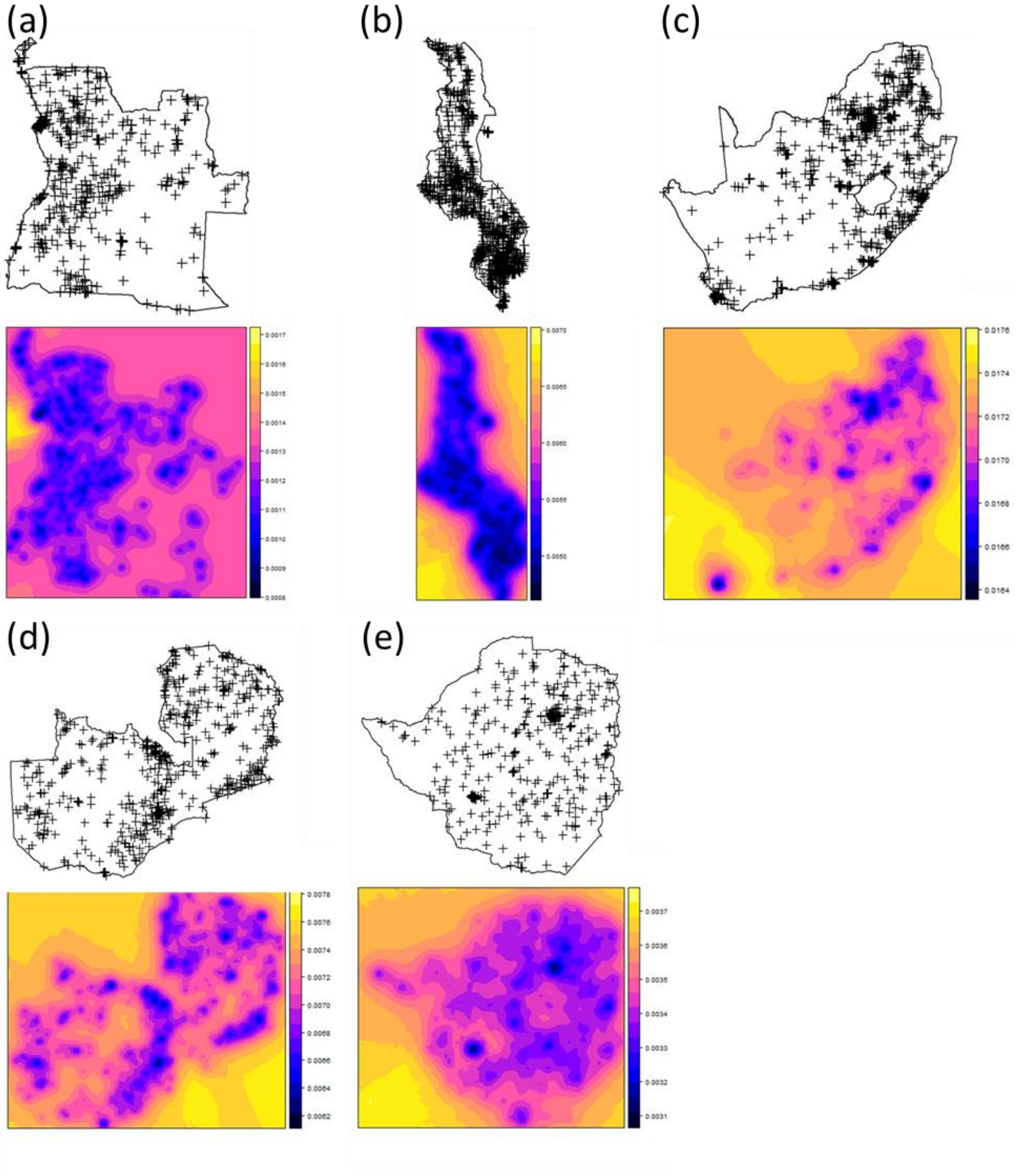

For each bivariate map, a bivariate palette has been designed to visually depict mobile individuals' proportion and HIV prevalence in all countries. Each variable has been normalized and separated with an equal interval (Tertile) data classification type. In figure A3.3, the lowest value for both variables become A1, while the cell C3 represents the max for both Migration percent and HIV prevalence. Any combination of the two variables can be identified by its position in the color scheme. We now have a proper color scheme that we can use as categories to symbolize both continuous surfaces. Noteworthy is that extreme rates in bivariate maps could be misleading and insufficiently reliable due to some PSUs with small populations. Hence, map results should be interpreted with caution. R programming environment (24) and packages including: *gstat* (28, 29), *raster* (30), and *leaflet* (31) were used to generate all maps.

Figure C3.3. Tertile bivariate color scheme

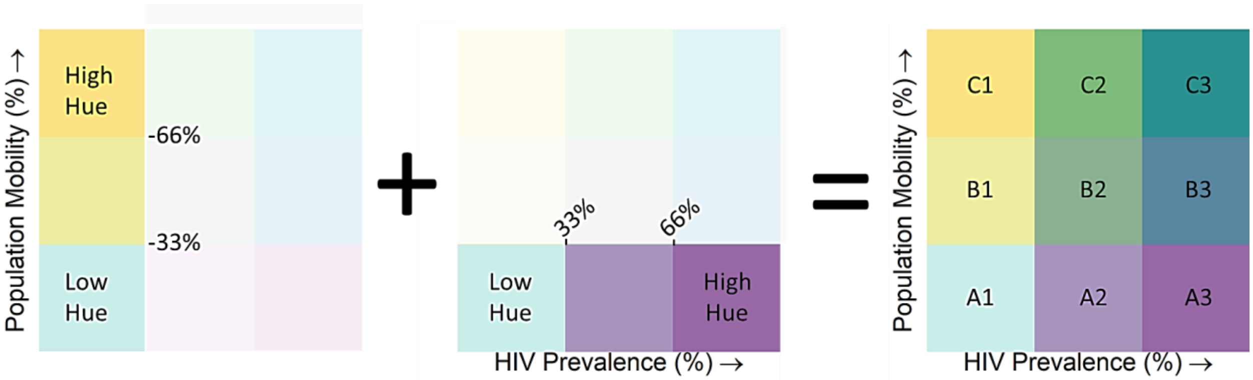

REFERENCES

1. Deane KD, Parkhurst JO, Johnston D; Linking migration, mobility and HIV. *Tropical Medicine & International Health* 2010;**15**(12):1458-1463. doi: 10.1111/j.1365-3156.2010.02647.x.

2. Mincer J; Family Migration Decisions. *Journal of Political Economy* 1978;**86**(5):749-773.

3. Voeten HACM, Vissers DCJ, Gregson S, et al.; Strong association between in-migration and HIV prevalence in urban sub-Saharan Africa. *Sexually transmitted diseases* 2010;**37**(4):240-243. doi: 10.1097/OLQ.0b013e3181c3f2d0.

4. Jochelson K, Mothibeli M, Leger J-P; Human Immunodeficiency Virus and Migrant Labor in South Africa. *International Journal of Health Services* 1991;**21**(1):157-173. doi: 10.2190/11ue-l88j-46hn-hr0k.

5. Glynn JR, Pönnighaus J, Crampin AC, et al.; The development of the HIV epidemic in Karonga District, Malawi. *AIDS* 2001;**15**(15):2025-2029.

6. Kate Grabowski M, Lessler J, Bazaale J, et al.; Migration, hotspots, and dispersal of HIV infection in Rakai, Uganda. *Nature Communications* 2020;**11**(1):976. doi: 10.1038/s41467-020-14636-y.

7. Camlin CS, Akullian A, Neilands TB, et al.; Population mobility associated with higher risk sexual behaviour in eastern African communities participating in a Universal Testing and Treatment trial. *Journal of the International AIDS Society* 2018;**21**(S4):e25115. doi: 10.1002/jia2.25115.

8. Camlin CS, Akullian A, Neilands TB, et al.; Gendered dimensions of population mobility associated with HIV across three epidemics in rural Eastern Africa. *Health & Place* 2019;**57**:339-351. doi: https://doi.org/10.1016/j.healthplace.2019.05.002.

9. Coffee MP, Garnett GP, Mlilo M, et al.; Patterns of Movement and Risk of HIV Infection in Rural Zimbabwe. *The Journal of Infectious Diseases* 2005;**191**(Supplement_1):S159-S167. doi: 10.1086/425270.

10. Brockerhoff M, Eu H; Demographic and Socioeconomic Determinants of Female Rural to Urban Migration in Sub-Saharan Africa. *The International Migration Review* 1993;**27**(3):557-577. doi: 10.2307/2547100.

11. Coast E; Local understandings of, and responses to, HIV: Rural–urban migrants in Tanzania. *Social Science & Medicine* 2006;**63**(4):1000-1010. doi: https://doi.org/10.1016/j.socscimed.2006.03.009.

12. Anglewicz P; Migration, marital change, and HIV infection in Malawi. *Demography* 2012;**49**(1):239-265. doi: 10.1007/s13524-011-0072-x.

13. Carswell JW, Lloyd G, Howells J; Prevalence of HIV-1 in east African lorry drivers. *AIDS* 1989;**3**(11):759-762.

14. Staveteig S, Head SK, Croft TN, et al.; Factors associated with prior testing among HIV-positive adults in Sub-Saharan Africa. *DHS Comparative Reports No. 43*. Rockville, Maryland, USA: ICF International, 2016.

15. UNAIDS/WHO; Consultation on STD interventions for preventing HIV: what is the evidence? *UNAIDS BEST PRACTICE COLLECTION*. Geneva, Switzerland: UNAIDS/WHO, 2000.

16. Akullian A, Bershteyn A, Jewell B, et al.; The missing 27. *AIDS (London, England)* 2017;**31**(17):2427-2429. doi: 10.1097/QAD.0000000000001638.

17. Cuadros DF, Li J, Mukandavire Z, et al.; Towards UNAIDS Fast-Track goals: targeting priority geographic areas for HIV prevention and care in Zimbabwe. *AIDS* 2019;**33**(2):305-314. doi: 10.1097/qad.0000000000002052.

18. Schaefer R, Gregson S, Eaton JW, et al.; Age-disparate relationships and HIV incidence in adolescent girls and young women: evidence from Zimbabwe. *AIDS* 2017;**31**(10):1461-1470. doi: 10.1097/qad.0000000000001506.

19. Zuma K, Gouws E, Williams B, et al.; Risk factors for HIV infection among women in Carletonville, South Africa: migration, demography and sexually transmitted diseases. *International Journal of STD & AIDS* 2003;**14**(12):814-817. doi: 10.1258/095646203322556147.

20. Sandefur GD, Scott WJ; A dynamic analysis of migration: an assessment of the effects of age, family and career variables. *Demography* 1981;**18**(3):355-368. doi: 10.2307/2061003.

21. Reniers G; Divorce and Remarriage in Rural Malawi. *Demographic Research* 2003;**S1**:175-206.

22. Camlin CS, Snow RC, Hosegood V; Gendered Patterns of Migration in Rural South Africa. *Population, Space and Place* 2014;**20**(6):528-551. doi: 10.1002/psp.1794.

23. Mayala B, Fish TD, Eitelberg D, et al.; The DHS Program Geospatial Covariate Datasets Manual. Rockville, Maryland, USA: ICF, 2018.

24. R Core Team; R: A Language and Environment for Statistical Computing. Vienna, Austria: R Foundation for Statistical Computing, 2018.

25. Lumley T; survey: analysis of complex survey samples. 2019.

26. Philip GM, Watson DF; A Precise Method for Determining Contoured Surfaces. *The APPEA Journal* 1982;**22**(1):205-212. doi: https://doi.org/10.1071/AJ81016.

27. Stein ML. *Interpolation of spatial data.* New York: Springer-Verlag, 1999.

28. Pebesma EJ; Multivariable geostatistics in S: the gstat package.

29. Gräler. B, Pebesma E, Heuvelink G; Spatio-Temporal Interpolation using gstat. *The R Journal 8*, 2016.

30. Hijmans RJ; raster: Geographic Data Analysis and Modeling. 2019.

31. Cheng J, Karambelkar B, Xie Y; leaflet: Create Interactive Web Maps with the JavaScript 'Leaflet' Library. 2018.
